# Effects of Cognitive Training in Mild Cognitive Impairmentmeasured by Resting State Functional Imaging

**DOI:** 10.3390/bs10110175

**Published:** 2020-11-17

**Authors:** Seungho Kim, Eunhee Park, Hyunsil Cha, Jae-Chang Jung, Tae-Du Jung, Yongmin Chang

**Affiliations:** 1 Department of Medical & Biological Engineering, Kyungpook National University, Daegu 41944, Korea; seungho5335@gmail.com (S.K.); hscha1002@daum.net (H.C.); 2 Department of Rehabilitation Medicine, School of Medicine, Kyungpook National University, Daegu 41944, Korea; ehmdpark@naver.com (E.P.); teeed0522@daum.net (T.-D.J.); 3 Department of Biology, College of Natural Sciences, Kyungpook National University, Daegu 41566, Korea; jcjung@knu.ac.kr; 4 Department of Radiology, Kyungpook National University Hospital, Daegu 41944, Korea; 5 Department of Molecular Medicine, School of Medicine, Kyungpook National University, Daegu 41944, Korea

**Keywords:** cognitive impairment, cognitive training, resting-state functional magnetic resonance imaging, functional connectivity

## Abstract

Mild cognitive impairment (MCI) is defined as an intermediate state of cognitive alteration between normal aging and dementia. In this study, we performed a functional network connectivity analysis using resting-state functional magnetic resonance imaging to investigate the association between changes in functional connectivity in the brain and the improvement in cognitive abilities after cognitive training. A computerized cognitive training program was used to improve the abilities of fifteen participants with MCI. The cognitive training program (Comcog), which consists of three weekly sessions totaling 90 min, was conducted with all participants over six weeks. The cognitive abilities before (pre-Comcog) and after (post-Comcog) the cognitive training process were measured using a neurocognitive function test. After the Comcog, the participants enhanced their visual and verbal memories, attention, and visuo-motor coordination. The functional connectivity between cingulo-opercular (CON) and default mode (DMN) showed significant improvements after Comcog training. Therefore, our study suggests that cognitive training may improve the cognitive abilities of participants. This improvement was associated with an increase in the functional connectivity between DMN and CON. The increase in functional connectivity after cognitive training was specifically associated with overall cognitive functions, including executive, memory, decision-making, and motivational functions.

## 1. Introduction

Mild cognitive impairment (MCI) is defined as an intermediate state of cognitive alteration between normal aging and dementia [1,2]. Cognitive impairment increases the risk of various diseases that are linked with dementia, such as Alzheimer’s and Parkinson’s, as well as other psychiatric symptoms, by accelerating cognitive dysfunction [3] and leading to a decline in the patients’ quality of life [4,5]. Fifteen to twenty percent of people over 65 years of age have MCI and, among these, fifteen percent develop dementia [6]. Cognitive training has been one of the solutions to reduce the risk of dementia or MCI because it improves patients’ cognitive abilities or delays the onset of symptoms [7,8]. However, there were recent reports stating that cognitive training has no effect on cognitive ability such as decision-making [9,10]. Therefore, the effect of cognitive training is still controversial.

Although this controversy exists, computerized cognitive training (CCT) programs have been increasingly used to improve cognitive functions [11,12]. CCT programs involve structured practice tasks and have proved to be effective in improving cognitive abilities such as language, visuo-spatial memory, and attention skills [13,14]. In addition, a previous meta-analysis of neuroimaging studies revealed that CCT prompted changes in brain activity or connectivity [15,16,17]. In addition, these studies identified the dysfunction of the default mode (DMN), cingulo-opercular (CON), and fronto-parietal network (FPN), components known as the “core of cognitive processing” [18,19], as the main factor influencing cognitive impairment [20,21]. In particular, older adults with MCI have DMN [22,23,24], which is responsible for performing verbal episodic memory and serving visuo-spatial attention functions [25,26,27], and FPN, which is responsible for initiating and adjusting overall cognitive control [28,29]. The CON, responsible for sustaining or updating internal attention settings, is also known to suffer dysfunction due to cognitive impairment [30,31].

In this study, we used resting-state functional magnetic resonance imaging (fMRI) to investigate what the effects of CCT are on the alteration of the resting-state functional connectivity among brain functional systems in participants with MCI. In particular, we used functional connectivity analysis, which is an increasingly used tool to observe associations among regions within or between brain networks [32]. Several functional connectivity studies have previously investigated the changes in cognitive functions using CCT in MCI patients. Despite these advances, it is still unclear what the effects of CCT are on the alteration of the resting-state functional connectivity among DMN, CON, and FPN in older adults with MCI. We focused on the changes in functional connectivity in DMN, CON, and FPN brain networks before and after CCT. We hypothesized that the CCT increases the cognitive abilities of participants by changing the functional connectivity between brain networks.

## 2. Materials and Methods

### 2.1. Participants

Fifteen MCI participants (mean age = 74.3 ± 5.83 years; mean education = 6.0 ± 3.113) were recruited in this study. These participants were determined as MCI patients by considering the following inclusion criteria: a < 1 score on the Korean version of theclinical dementia rating (CDR) [33]; a > 18 score on the mini-mental state examination (MMSE) based on the Korean version of the Consortium to Establish a Registry for Alzheimer’s Disease (CERAD-K) assessment packet [34,35,36]; and no history of brain injury or brain diseases such as degenerative or psychiatric disorders. Written informed consent was obtained from each participant and ethical approval was provided by the Institutional Review Board of Kyungpook National University Chilgok Hospital.

### 2.2. Computerized Cognitive Training (CCT) System

All participants performed the cognitive training using the Comcog system (Maxmedica, Seoul, Korea) for six weeks. The training course consisted of three weekly sessions totaling 90 min. The Comcog, which is commonly used in Asia [37], was used as a CCT system. The system was composed of ten activities for cognitive training: two visual and two auditory tasks that evaluate the response time to visual and auditory stimuli, respectively; two attention tasks that track attention during distraction periods; three working memory tasks that evaluate recognition and recall memory upon visual, auditory, and multisensory stimuli; and one emotional task that evaluates responses upon an emotional stimulus.

### 2.3. Neurocognitive Measurements

All participants had their cognitive function assessed using CDR, MMSE, and a computerized neurocognitive function test (CNT; Maxmedica, Seoul) one week before CCT as pre-test and one week after CCT as post-test. All tests were conducted by an occupational therapist, who completed the required certification program. The CDR and MMSE have been used in testing cognitive performances in clinical settings and to synthetically evaluate the orientation, attention, judgment, memory, language, and visuo-spatial functions. The CNT is also a method for evaluating degrees of cognitive impairment [38,39]; it consists of six categories, including a verbal memory test, visual memory test, attention test, visuo-motor coordination test, and high cognition test. These tests were distributed in fourteen sessions: 4 sessions of verbal memory tests; 4 sessions of visual memory tests; 2 sessions of attention tests; 2 sessions of visuo-motor coordination tests, and 2 sessions of high cognition tests.

### 2.4. Image Acquisition and Analysis

A Discovery MR750w 3.0T scanner (GE healthcare, Milwaukee) with a 24-channel head coil was used to obtain all structural and functional imaging data. A 3D brain volume imaging (BRAVO) sequence was used to obtain structural images (echo time (TE) = 3.2 ms, repetition time (TR) = 8.4 ms, field of view (FOV) = 25.6 cm, matrix = 256 × 256, slice thickness = 1 mm, flip angle (FA) = 12 degrees). A T2*-weighted gradient echo planar imaging pulse sequence was used to obtain functional images (TE = 30 ms, TR = 2000 ms, FOV = 23 cm, matrix = 64 × 64, slice thickness = 4 mm, FA = 90 degrees).

All functional images were preprocessed using a statistical parametric mapping toolbox (SPM12; http://www.fil.ion.ucl.ac.uk/spm). The slice-timing correction, realignment, and co-registration were included in the preprocessing steps for temporal, spatial interpolation, and motion correction, respectively. All functional data were normalized to an MNI standard space. A functional connectivity toolbox (CONN; https://web.conn-toolbox.org/) was used to process the functional connectivity analysis. Individual images were denoized to minimize the influence of artifactual factors. The denoizing steps included temporal band-pass filtering (0.008–0.09 Hz) and linear regression (motion, white matter, cerebrospinal fluid (CSF), and outlier scan effects).

Region of interest (ROI)-to-ROI correlation analysis was applied to investigate the functional connectivity among ROIs associated with cognitive systems. The significance of the difference in the functional connectivity between the two conditions (pre-Comcog vs. post-Comcog) was determined by a paired *t*-test. The total ROI consisted of 87 spheres with 5 mm radii each, which were included in the DMN, CON, and FPN based on Dosenbach 160 functional ROIs [40]. Specifically, six specific regions were included within the DMN: right precuneus, left posterior cingulate cortex (PCC), right superior frontal gyrus (SFG), bilateral ventro-medial prefrontal cortex (vmPFC), and medial prefrontal cortex (mPFC). The CON contained a total of ten regions, which include the dorsal (dACC) and middle (ACC) part of anterior cingulate cortex, anterior (aINS) and posterior (pINS) part of insula (INS), ventro-lateral prefrontal cortex (vlPFC), orbito-frontal cortex (OFC), caudate, and thalamus.

The ROI-to-ROI connection between each condition was determined by the false discovery rate (FDR) corrected at the cluster level *p* < 0.05 and uncorrected *p* < 0.05 at the connection level (post-hoc) based on functional network connectivity multivariate parametric statistics [41]. The REX toolbox (https://www.nitrc.org/projects/rex) was used to extract connection values from ROIs.

### 2.5. Statistical Analysis

The statistical package for social sciences (SPSS 25; http://www.ibm.com/analytics/spss-statistics-software) was used for statistical analysis. According to the assumption of normality of variances, a paired *t*-test or Wilcoxon signed-rank test were performed between pre- and post-Comcog from neurocognitive measurements to evaluate the change in cognitive abilities. Sensitivity analyses were performed for all neurocognitive measurements using g*power (http://www.gpower.hhu.de) for preventing statistical errors [41]. The alpha and beta error probabilities were defined as 0.05 and 0.2, respectively, to assess the appropriate effect size (d = 0.675) on fifteen samples. All effect size factors were calculated using the mean, standard deviation, and correlation between groups. The correlation analysis was performed on the differences between each condition in the functional connection values of ROIs and cognitive abilities. For the correlation analysis, a partial correlation analysis was performed using SPSS 25, after removing the Beck depression inventory (BDI) effects.

## 3. Results

### 3.1. Neurocognitive Abilities

As a result of the normality test, 7 out of 17 sessions, consisting of CNT, MMSE, CDR, and BDI, could assume normality: MMSE; digit span B; verbal delayed recall; visual span A/B; visual delayed recall; auditory CPT. There were significant differences between the pre- and post-Comcog conditions in the CDR (z = 2.828, *p* = 0.008, d = 1.653) and MMSE scores (t = 6.012, *p* = 0.000, d = 1.915), which assess cognitive dysfunction. In addition, we observed an improvement in cognitive function after 14 sessions of CNT in the post-Comcog condition. Detailed cognitive measures are summarized in Table 1.

### 3.2. Functional Connectivity and Correlation Analysis

**Figure 1:**
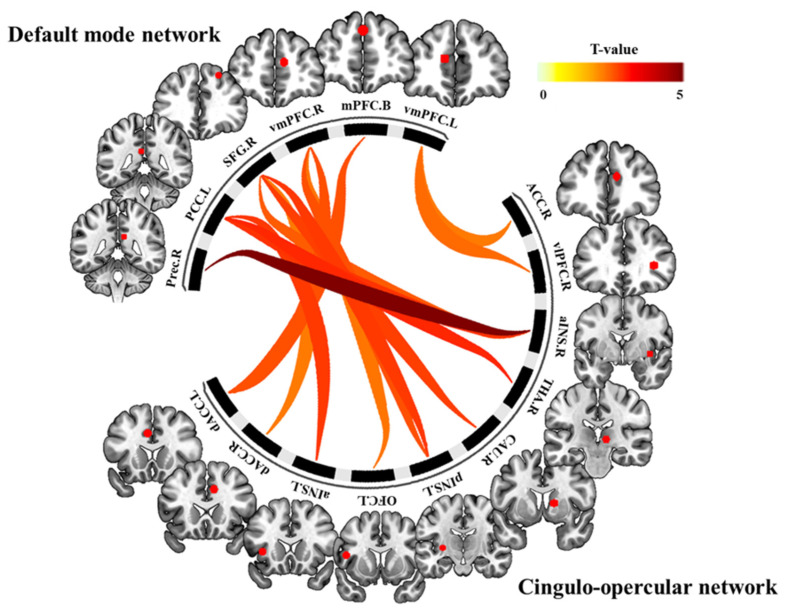
The functional connectivity between cingulo-opercular (CON) and default mode (DMN) showed significant differences between the pre- and post-Comcog conditions in the paired *t*-test (false discovery rate (FDR) < 0.05 corrected in cluster level). Fourteen positive connections between regions of interest (ROIs) in CON and DMN showed improved connections in the post-Comcog condition.

**Figure 2:**
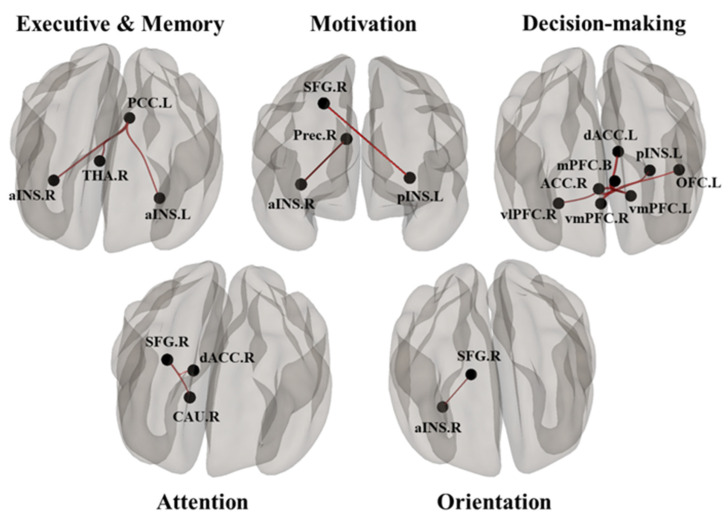
The functional connections between ROIs in CON and DMN, which showed high correlations with improved cognitive functions (executive and memory, motivation, decision-making, attention), as revealed by neurobehavioral tests.

DMN and CON showed significant differences between the pre- and post-Comcog conditions in the paired *t*-test (FDR < 0.05 corrected in cluster level) (Figure 1). Specifically, there were fourteen positive connections between ROIs in the paired *t*-test. These positive connections mean improved connections in the post-Comcog condition. For example, the right precuneus was functionally connected with the right aINS and the left PCC showed a significant increase in functional connectivity with the bilateral aINS and the thalamus. In addition, we observed positive differences in connections between the right SFG and the left pINS, right aINS, dACC, and caudate. Notably, the mPFC was functionally connected with the left dACC and the left side of the vmPFC showed an enhanced functional connectivity with the right ACC and vlPFC. Additionally, the right side of the vmPFC was connected with the left dACC, OFC, and pINS (Figure 1 and Figure 2, Table 2).

**Figure 3:**
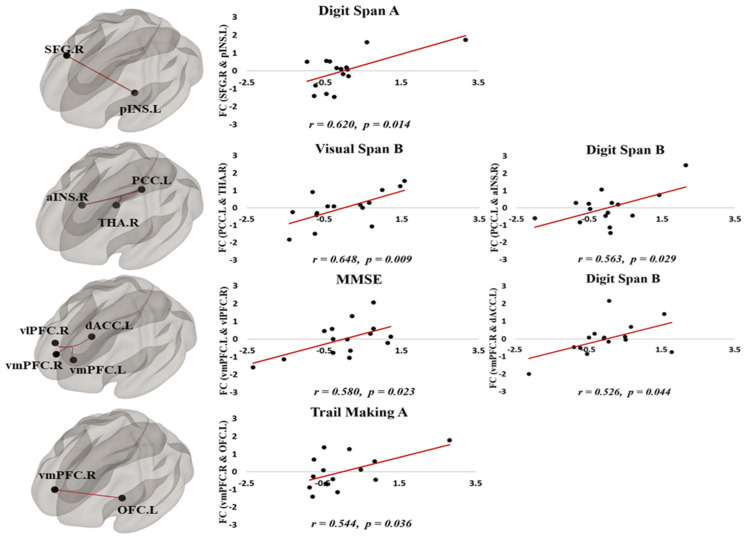
The functional connectivity among ROIs was associated with neurocognitive abilities after CCT training. In the partial correlation analysis, the connectivity between brain structures in core cognitive brain networks was positively correlated with neurobehavioral test scores.

The functional connectivity among ROIs was associated with neurocognitive abilities. In the partial correlation analysis, the left vmPFC–right vlPFC connectivity was positively correlated with the MMSE score (r = 0.580, *p* = 0.023) and the right SFG–left pINS connectivity showed a positive correlation with the digit span A score (r = 0.620, *p* = 0.014), which is associated with verbal memory function. In addition, the functional connections on the left PCC–right aINS and right vmPFC–left dACC showed significant correlations with the digit span B score (r = 0.563, *p* = 0.029 and r = 0.526, *p* = 0.044 respectively). Moreover, the left PCC–right thalamus connectivity had a positive correlation with the visual span B score (r = 0.648, *p* = 0.009), which is associated with visual memory ability. Finally, the right vmPFC–left OFC connectivity was correlated with the trail-making A test (r = 0.544, *p* = 0.036), which is associated with visuo-motor coordination. Detailed results are summarized in Figure 2 and Figure 3 and in Table 2.

## 4. Discussion

The current study shows that an increase in brain functional connectivity after cognitive training may improve the cognitive abilities of MCI patients. In particular, the improvement in the functional connectivity between the DMN and CON was closely associated with enhanced cognitive functions, including executive, memory, decision-making, and motivation functions. Although several neuroimaging studies have already reported the effects of CCT on the brain activity of older adults [16,17,42], the interpretation of these effects on the alteration of resting state, functional network connectivity among DMN, CON, and FPN in MCI patients has thus far not been conclusive.

From our neurobehavioral analysis, we conclude that, after six weeks of cognitive training using the CCT system, participants showed an improvement in their verbal short-term and long-term memory abilities, with increased scores in digit span A/B and verbal delayed recall tests. In addition, participants showed significant improvements in visual span B and visual delayed recall tests, which assess the visual short-term and long-term memory functions. The participants also showed increased scores in the trail-making test, which evaluates the visuo-motor coordination function.

The improved cognitive functions revealed by the neurobehavioral tests were highly correlated with alterations in the functional connectivity between DMN and CON (Figure 2). From the functional connectivity analysis, the INS regions showed increased connectivity with PCC/precuneus, SFG, and vmPFC after 6 weeks of cognitive training. The pINS–SFG connectivity showed a positive correlation with the digit span A test, suggesting an improvement in motivational ability after cognitive training in our study. This result was consistent with those of a previous study where the increase in motivational ability improved participants’ performance in the digit span test [43]. In addition, the aINS–PCC connectivity was positively correlated with the scores in the digit span B test, which evaluates the verbal short-term memory function. Because the aINS–PCC connections were positively correlated with the executive and memory functions [44,45], we propose that cognitive training may strengthen the connectivity between these brain structures and thus improve the executive and memory functions. We also showed that the pINS–SFG connectivity positively correlates with the scores in the digit span A test. Because the pINS–SFG connections were previously linked to motivational ability [46,47], our result is consistent with the observation that an increased motivational ability can improve the performance on the digit span test [43].

After CCT training, the mPFC and vmPFC regions also showed an increased connectivity with the ACC and OFC regions, respectively. In addition, the connectivity of vmPFC–vlPFC, PCC–thalamus, SFG–daCC, and SFG–caudate increased after training. The vmPFC–vlPFC connectivity was correlated with the MMSE score, whereas the vmPFC–daCC connectivity was correlated with the digit span B scores. These correlations suggest that an improvement in comprehension and judgment is the reason for a higher MMSE score and that the increase in the ability to integrate information positively affects the verbal short-term memory function. The PCC–thalamus connectivity was positively correlated with the scores in the visual span B test, which is related to visual short-term memory function. Because the PCC–thalamus connections were previously associated with semantic memory, executive, and visuo-spatial functions [20,48], the increased positive correlation between these two regions upon CCT suggests that cognitive training may strengthen their connectivity, resulting in improved executive and visuo-spatial functions.

Notably, our study contains some limitations. First, the sample size (*n* = 15) is small. Future studies measuring the resting functional connectivity in larger sample sizes are warranted to support the findings of the present study. Second, the association between neurobehavioral performance and functional connectivity was extrapolated by taking into consideration a correlation analysis. In future studies, more detailed analyses are required to investigate the suggested association between behavioral and functional imaging measurements. Third, participants performed cognitive training for six weeks only, which is not enough for investigating dynamic changes in brain connectivity. We thus need to expand the current study to longer periods to investigate the permanent effect of CCT on cognitive ability and brain functional connectivity. Finally, in this study, there was no active control group. Therefore, the performance gains shown in participants should be considered as relative gains and the lack of an active control group should be noted as a limitation of this study.

## 5. Conclusions

We observed that cognitive training in participants with MCI increased the brain functional connectivity between different brain networks that are strongly associated with cognitive functions. In particular, the increase in functional connectivity between DMN and CON was closely associated with neurobehavioral improvements, including executive, memory, decision-making, and motivation functions. Therefore, our findings suggest that cognitive training may strengthen the connectivity between core cognitive brain structures and that this can, in turn, improve their associated cognitive functions.

## Figures and Tables

**Table 1:** Demographic and neurocognitive characteristics of participants with mild cognitive impairment before and after Comcog training.

Participants (N = 15)	Pre-Comcog	Post-Comcog	Paired *t*-Test (t)	Wilcoxon Signed-Rank Test (z)	Effect Size (d)
Age (years)	74.3 ± 5.83				
Education (years)	6.0 ± 3.113				
BDI	9.6 ± 8.10	10.3 ± 8.23		−0.420	0.082
CDR	0.8 ± 0.25	0.5 ± 0.37		−2.828 **	1.186 ^†^
MMSE	24.3 ± 3.08	26.9 ± 2.57	6.011 *		1.513 ^†^
CNT (t-value)					
Verbal memory test					
Digit span A	32.1 ± 5.28	39.9 ± 11.62		−3.197 **	0.951 ^†^
Digit span B	36.4 ± 6.86	45.1 ± 7.77	3.708 **		0.954 ^†^
Verbal learning	28.7 ± 3.47	29.3 ± 3.97		−0.535	0.225
Verbal delayed recall	30.9 ± 5.49	36.6 ± 7.98	3.332 *		0.866 ^†^
Visual memory test					
Visual span A	34.1 ± 9.44	37.5 ± 9.12	1.379		0.363
Visual span B	33.3 ± 7.56	37.8 ± 7.31	2.802 *		0.718 ^†^
Visual learning	37.9 ± 11.44	41.9 ± 11.48		−2.804 **	0.669
Visual delayed recall	46.2 ± 7.94	51.4 ± 8.29	3.948 **		1.021 ^†^
Attention test					
Visual CPT	49.1 ± 19.25	56.2 ± 18.67		−2.366 *	0.653
Auditory CPT	48.9 ± 18.23	51.6 ± 14.27	1.286		0.335
Visuo-motor coordination					
Trail-making A	41.3 ± 11.15	46.7 ± 12.34		−2.810 **	0.775 ^†^
Trail-making B	43.8 ± 15.28	49.0 ± 19.61		−2.324 *	0.602
High cognition test					
Card sorting test	54.9 ± 22.96	62.5 ± 15.49		−2.197 *	0.543
Word color test	35.6 ± 11.38	41.4 ± 14.08		−2.207 *	0.570

BDI, Beck depression inventory; CDR, clinical dementia rating scale; MMSE, mini-mental state examination; CNT, computerized neurocognitive function test. All neurocognitive measurements, except BDI and CDR in the Wilcoxon signed-rank test, were based on a negative rank. * *p* < 0.05, ** *p* < 0.01, ^†^ d > 0.675.

**Table 2:** The functional connectivity between ROIs for a paired *t*-test in participants with mild cognitive impairment (MCI) (post-Comcog–pre-Comcog condition).

Comparison	Regions in Network		Left/Right	Coordinates (mm)			Connectivity (T-Value)
	DMN	CON		x	y	z	
Post > Pre	Precuneus		R	9	−43	25	
		aINS	R	37	−2	−3	4.73
	PCC		L	−5	−43	25	
		aINS	L	−36	18	2	2.68
		aINS	R	37	−2	−3	2.73
		Thalamus	R	11	−24	2	2.83
	SFG		R	23	33	47	
		pINS	L	−30	−14	1	2.70
		aINS	R	37	−2	−3	2.41
		dACC	R	9	20	34	2.16
		Caudate	R	14	6	7	2.67
	mPFC		B	0	51	32	
		dACC	L	−6	17	34	2.49
	vmPFC		L	−11	45	17	
		ACC	R	9	39	20	2.15
		vlPFC	R	34	32	7	2.28
	vmPFC		R	9	51	16	
		dACC	L	−6	17	34	2.50
		OFC	L	−46	10	14	2.22
		pINS	L	−30	−14	1	2.53

FDR < 0.05 corrected in cluster level, and uncorrected < 0.05 in connection level. DMN, default mode network; CON, cingulo-opercular network; aINS, anterior-insula; pINS, posterior-insula; SFG, superior frontal gyrus; dACC, dorsal anterior cingulate cortex; ACC, anterior cingulate cortex; mPFC, medial prefrontal cortex; vmPFC, ventro-medial prefrontal cortex; vlPFC, ventro-lateral prefrontal cortex; OFC, orbito-frontal cortex.
